# A newborn with ankyloblepharon filiforme adnatum: a case report

**DOI:** 10.4076/1757-1626-2-8146

**Published:** 2009-08-14

**Authors:** Anna Maria Gruener, Manjit S Mehat

**Affiliations:** 1 Department of Ophthalmology, South London Health Care NHS Trust, Queen Mary's Hospital, Frognal Avenue, Sidcup, Kent DA14 6LT, UK; 2 Department of Ophthalmology, University Hospital North Staffordshire, Stoke-on-Trent, Staffordshire, ST4 7LN, UK

## Abstract

Ankyloblepharon filiforme adnatum is a rare congenital anomaly. We report a case of ankyloblepharon filiforme adnatum in a newborn Caucasian male whose paediatric examination was otherwise unremarkable. Ankyloblepharon filiforme adnatum can present as an isolated finding, in association with other anomalies, or as part of a well-defined syndrome.

## Introduction

The developing eyelid margins remain fused until the fifth month of gestation, but may not be completely separated until the seventh month [1]. Ankyloblepharon filiforme adnatum (AFA) is a rare benign congenital anomaly characterised by partial or complete full thickness fusion of the lid margins.

## Case presentation

**Figure 1:**
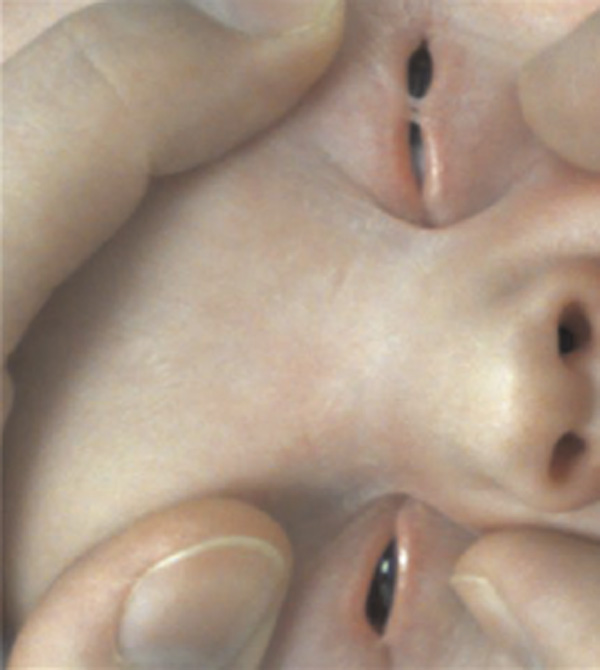
**Photograph showing several extensile bands of tissue connecting the eyelid margins of the right and left eye**.

A Caucasian male was born at 40 weeks' gestation by spontaneous vaginal delivery. The pregnancy was uneventful and the mother denied taking any drugs. The baby had bilateral partially fused eyelids at birth; paediatric assessment failed to identify any other congenital abnormalities. Ophthalmic examination revealed multiple fine bands of elastic tissue connecting the upper and lower lid margins (Figure 1). The elastic adhesions were divided with a suture cutter without any bleeding. The posterior surfaces of the eyelids appeared normal, as were ocular movement, anterior segment and fundus examination. Neither sedation nor local anaesthetic was necessary.

## Discussion

AFA was first described by Josef von Hasner in 1881, and is characterised by partial or complete adhesion of the ciliary edges of the upper and lower eyelids [2]. Usually, AFA constitutes a solitary malformation of sporadic occurrence. However, it can occur in an autosomal dominant pattern associated with cleft lip and palate. In addition, it has been reported in the context of trisomy 18 (Edwards' syndrome), Hay-Wells syndrome (a variant of the ectodactyly-ectodermal dysplasia-cleft lip palate syndrome), the popliteal pterygium syndrome (characterised by intercrural webbing of the lower limbs), and CHANDS (curly hair-ankyloblepharon-nail dysplasia). Other associations may include hydrocephalus, menigocoele, an imperforate anus, bilateral syndactyly, infantile glaucoma and cardiac problems such as patent ductus arteriosus and ventricular septal defects [3]. The aetiology of AFA is unknown, but failure of apoptosis at a critical stage in eyelid development has been suggested [4]. Timely separation of the eyelids is crucial to avoid the development of occlusion amblyopia.

This case report demonstrates the simplicity in treating AFA; it also highlights that its presence should alert the clinician to the possibility of an underlying congenital disorder.

## Conclusion

This case report demonstrates the simplicity in treating AFA; it also highlights that its presence should alert the clinician to the possibility of an underlying congenital disorder.

## Abbreviations

AFA: ankyloblepharon filiforme adnatum.

## Consent

Written informed consent was obtained from the patient's parent for publication of this case report and accompanying images. A copy of the written consent is available for review by the Editor-in-Chief of this journal.

## Competing interests

The authors declare that they have no competing interests.

## Authors' contributions

AMG was the major contributor in writing the manuscript. MSM contributed to editing the manuscript and accompanying image. All authors read and approved the final manuscript.
